# Molecular Origins of Long-Term Changes in a Competitive Continuous Biosensor with Single-Molecule Resolution

**DOI:** 10.1021/acssensors.4c00107

**Published:** 2024-07-05

**Authors:** Sebastian Cajigas, Arthur M. de Jong, Junhong Yan, Menno W. J. Prins

**Affiliations:** † Helia Biomonitoring, 5612 AR Eindhoven, The Netherlands; ‡ Department of Biomedical Engineering, Eindhoven University of Technology, 5612 AZ Eindhoven, The Netherlands; § Department of Applied Physics, Eindhoven University of Technology, 5612 AZ Eindhoven, The Netherlands; ∥ Institute for Complex Molecular Systems (ICMS), Eindhoven University of Technology, 5612 AZ Eindhoven, The Netherlands

**Keywords:** continuous biosensing, single-molecule sensor, long-term changes, molecular origins, aging studies

## Abstract

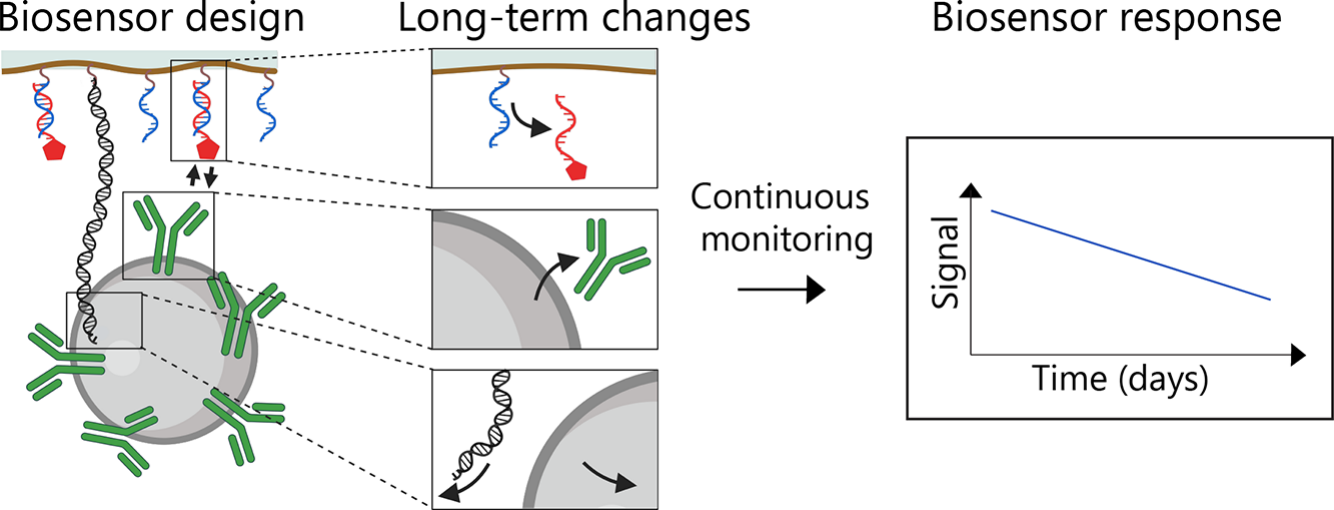

Biosensing by particle motion is a biosensing technology that relies on single-molecule interactions and enables the continuous monitoring of analytes from picomolar to micromolar concentration levels. However, during sensor operation, the signals are observed to change gradually. Here, we present a comprehensive methodology to elucidate the molecular origins of long-term changes in a particle motion sensor, focusing on a competitive sensor design under conditions without flow. Experiments were performed wherein only the particles or only the surfaces were aged in order to clarify how each individual component changes over time. Furthermore, distributions of particle motion patterns and switching activity were studied to reveal how particle populations change over timespans of several days. For a cortisol sensor with anticortisol antibodies on the particles and cortisol analogues on the sensing surface, the leading hypotheses for the long-term changes are (i) that the particles lose antibodies and develop nonspecific interactions and (ii) that analogue molecules dissociate from the sensing surface. The developed methodologies and the acquired insights pave a way for realizing sensors that can operate over long timespans.

Biosensing technologies for the continuous monitoring of biomolecules are being developed to enable monitoring and control functionalities in fields such as healthcare,^1,2^ industrial biotechnology, organ-on-chip research,^3,4^ industrial food processing,^5,6^ and environmental monitoring.^7^ Continuous sensors are commercially available for the continuous minimally invasive monitoring of glucose.^8^ However, glucose levels in both diabetic and healthy individuals are high, typically in the millimolar range.^9^ It remains a significant technological challenge to develop biosensors for the continuous monitoring of molecules at low concentrations (below millimolar). To enable continuous real-time biomolecular monitoring of a variety of analytes, affinity-based sensing principles are being investigated using, for example, electrochemical aptamer-based sensing,^10−13^ fluorescence-based sensing,^14−16^ and biosensing based on particle motion.^17−20^ Proofs of concept of continuous affinity-based sensing have been demonstrated for the monitoring of small molecules as well as macromolecules. However, only limited studies have addressed the long-term changes of affinity-based sensors over time scales of days.^12,13,21^

Biosensing by particle motion (BPM) is an affinity-based continuous biosensing method with hundreds to thousands of biofunctionalized particles that dynamically interact with a biofunctionalized surface. The particles switch between unbound states with high mobility and bound states with low mobility, due to affinity-based single-molecule interactions that are influenced by analyte molecules.^17,18,20,22^ The particle switching rate depends on the analyte concentration in solution and can be continuously measured using video microscopy.^19,22,23^ The sensing method does not consume or produce any reagents, which makes it suitable for long-term continuous sensing applications. In previous studies, BPM sensors functioned up to 24 h, and mathematical corrections were applied to compensate for signal changes over time.^6,18,20^ However, the origins of signal changes were not clear.

Here, we present an investigation into long-term changes in a BPM sensor, exemplified with cortisol as an analyte. Experimental methodologies were developed to study sensor changes over long time scales. To clarify the mechanisms underlying the signal changes, we separately aged particles and sensing surfaces under different conditions, whereafter BPM signals were measured with and without analyte in solution. Sensor changes were studied in terms of switching events, state lifetimes, and particle motion patterns. The results lead to hypotheses about the causes of long-term changes in the sensor. The developed experimental methodologies and the insights gained will support the development of affinity-based continuous biosensors that can operate over days and weeks.

## Results and Discussion

### Particle-Based Biosensor for Continuous Operation

**Figure 1:**
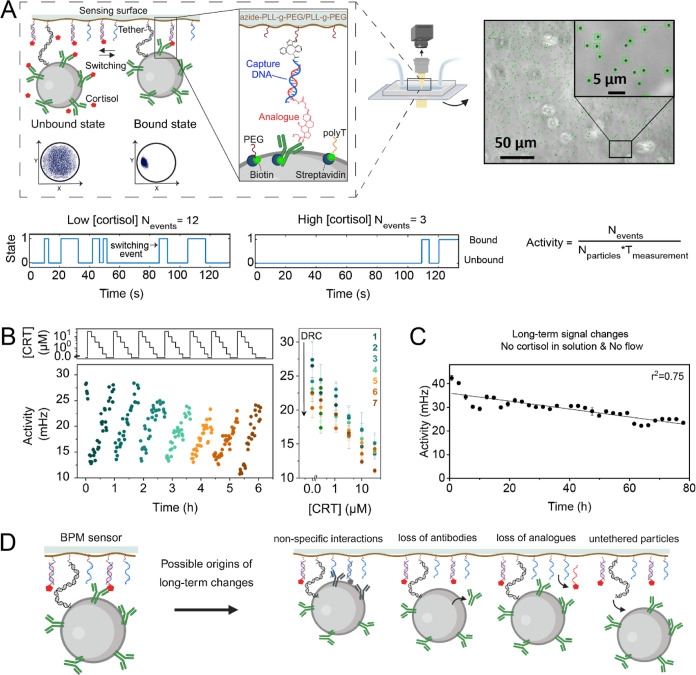
Schematic of a continuous monitoring sensor based on tethered particle motion (t-BPM) for measuring cortisol (CRT). (A) The left panel shows the design of a competition-based biosensor with tethered particles used in this study.^23^ The sensing surface is coated with a low-fouling polymer layer with azide-functionalized PLL-*g*-PEG bottle-brush polymers, to which DBCO-dsDNA tether molecules (black) and DBCO-ssDNA capture molecules (blue)^18^ are covalently coupled. Streptavidin-coated particles are functionalized with biotinylated antibodies and then coupled to the sensing surface via a dsDNA tether molecule with biotin functionalization (green dot). Cortisol analogue molecules (red) are coupled to the sensing surface by hybridization to the ssDNA capture molecules. The antibody–analogue interaction is reversible, which causes particles to switch between bound and unbound states. The example state–time traces at the bottom of the figure show that the switching rate between bound and unbound states is influenced by the presence of the analyte in solution. For more details, see Van Smeden et al.^23^ The particles are coupled to the top inner surface of a flow cell; see the sketch. The right panel shows more than 1000 particles that are continuously monitored and analyzed with video microscopy. Green squares show particles identified by the particle tracking algorithm. (B) Example of a cortisol biosensor that shows long-term changes. Six dose–response curves were sequentially measured on the same sensor. Each activity data point was measured during 1 min. The right panel shows the six dose–response curves derived from the data in the left panel. The error bars represent the standard deviations calculated from the 5 measurements obtained from the data shown in the left panel. (C) Sensor signal measured in static mode, i.e., in the absence of replacement of fluid, recorded for 78 h. No cortisol was present in the solution. Each data point is the mean of 10 measurements of 5 min. Error bars are the standard deviations of the 10 measurements. The linear fit gives a relative loss rate of the activity of 0.40 ± 0.04 % per hour. (D) Schematic representation of possible mechanisms that could cause long-term changes in the biosensor and that are investigated in this paper.

Long-term changes were investigated for a tethered biosensor based on particle motion (t-BPM) with a competitive format, as sketched in Figure 1A. Streptavidin-coated particles (Dynabeads MyOne) were functionalized with biotinylated anticortisol antibodies and partially blocked with biotinylated polyT molecules. The sensing surface was functionalized with a polymer layer (PLL-*g*-PEG/azide-PLL-*g*-PEG) to allow covalent immobilization by the click chemistry of DBCO-dsDNA-biotin tethers and DBCO-ssDNA capture molecules. The particles were tethered to the sensing surface via a dsDNA tether. After tethering, the remaining streptavidin binding sites on the particles were blocked with biotin-PEG to prevent multitethering and reduce nonspecific interactions between the particle and the sensing surface. At this stage, particles can move freely within the range the tether allows, and the particles have little interaction with the sensing surface. When the cortisol analogue was supplied (a cortisol-modified ssDNA),^23^ they hybridize to the ssDNA capture molecules on the sensing surface, enabling the formation of specific reversible bonds between antibodies on the particle and the cortisol analogue on the sensing surface. The transient binding between the antibody and the analogue causes particles to switch between bound and unbound states. The bottom panels of Figure 1A show examples of state transition traces for low and high analyte concentrations (see also Supporting Information SI1). At low concentrations of cortisol in solution, the switching between states occurs more frequently compared to high concentrations of cortisol analyte because cortisol molecules occupy binding sites on the antibodies. The state switching of the particles was observed by recording the motion of the particles with a bright-field microscope.^17,20^ Thousands of particles were observed at the same time, the particle positions were tracked using a particle tracking algorithm, the switching events were determined in the individual time traces, and then the average number of switching events per particle per unit of time was reported as the activity signal with unit mHz.^22,24^ In a competitive BPM sensor, the switching activity correlates inversely with the analyte concentration since higher analyte concentrations cause fewer switching events.

Figure 1B shows the continuous monitoring of cortisol in a sequence of dose–response curves, measured on a single BPM sensor with tethered particles, as illustrated in Figure 1A. The top-left panel displays the concentration–time profiles, with cortisol concentrations in the range from 0 to 30 μM, applied over a period of about 6 h. The sensor responds in an analyte-dependent manner, as increased cortisol concentrations lead to lower activity values; see the bottom-left panel. The right panel shows seven dose–response curves resulting from the data in the bottom-left panel. It appears that the maximum signals (at 0 nM analyte concentration) gradually decrease over time, from 28 to about 20 mHz over a time span of 6 h.

**Figure 2:**
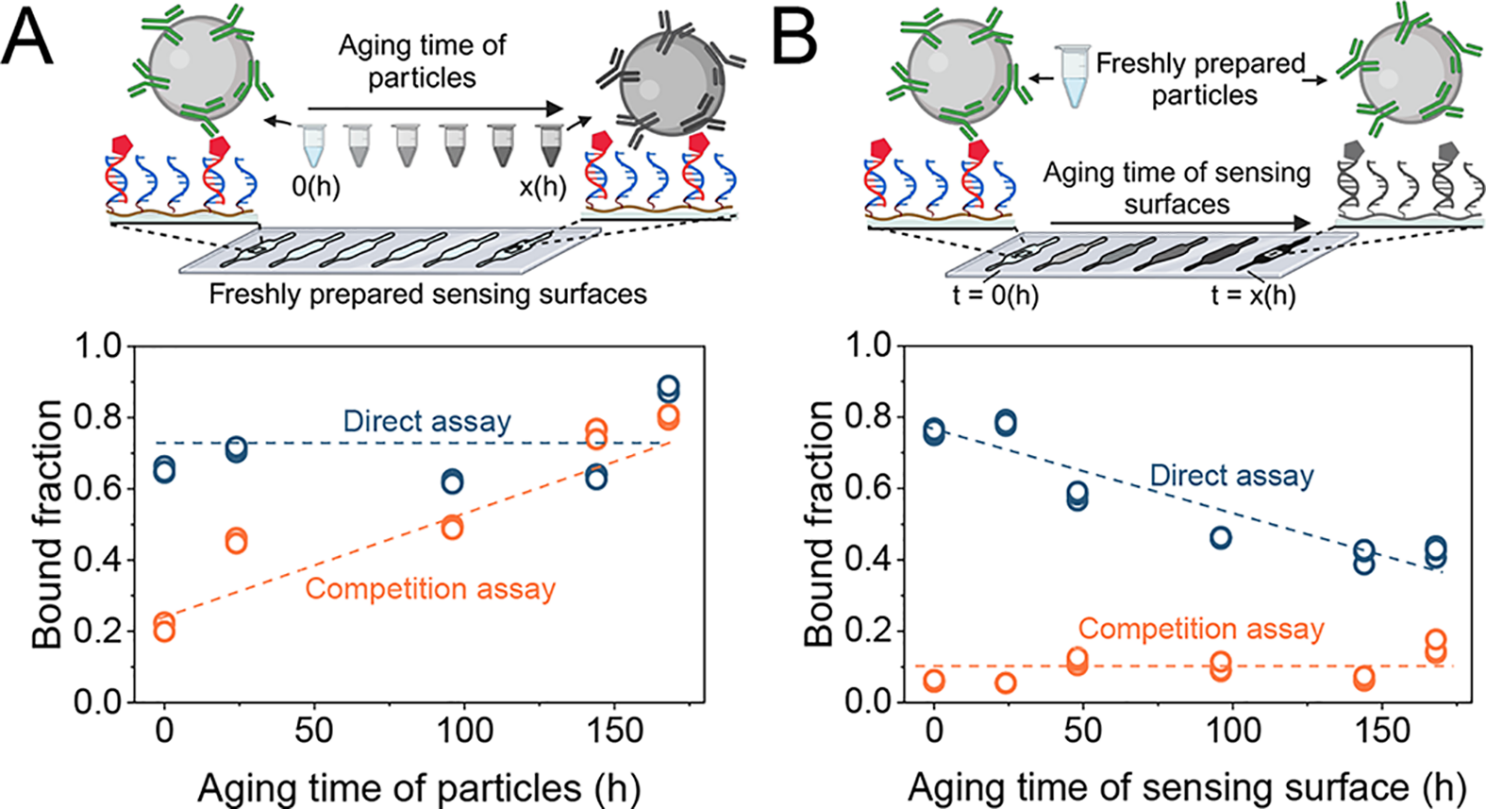
Study of separate particle aging and surface aging with readout using free particles on a sensing surface (f-BPM). Experiments were performed on polyethylene-derivative slides, each with 6 individual microfluidic channels. (A) Particles with anticortisol antibodies were prepared on different days, distributed over a period of 7 days. Particles were diluted 2500 times in 0.5 M NaCl in PBS at RT on a rotatory fin. On the last day, the differently aged particles were suspended in buffer without cortisol (direct assay, blue) or suspended in buffer with 30 μM of cortisol (competition assay, orange) and then added to sensing surfaces that were freshly prepared with 500 pM analogue.^19^ (B) Sensing surfaces with cortisol analogue were prepared on different days, distributed over a period of 7 days. After incubating the cortisol analogue solution (500 pM) for 1 h, the flow cells were flushed with 0.5 M NaCl in PBS. The sensing surfaces were then stored in 0.5 M NaCl in PBS at RT for long-term aging. The aging was performed in a humidity chamber to prevent drying of the fluidic channels. On the last day, all prepared sensing surfaces were studied with freshly prepared particles, suspended in buffer without cortisol (direct assay), or suspended in buffer with 30 μM cortisol (competition assay). Duplicate measurements were performed to evaluate the reproducibility of the aging experiments (see Figure S4). Dashed lines are guides for the eye.

**Figure 3:**
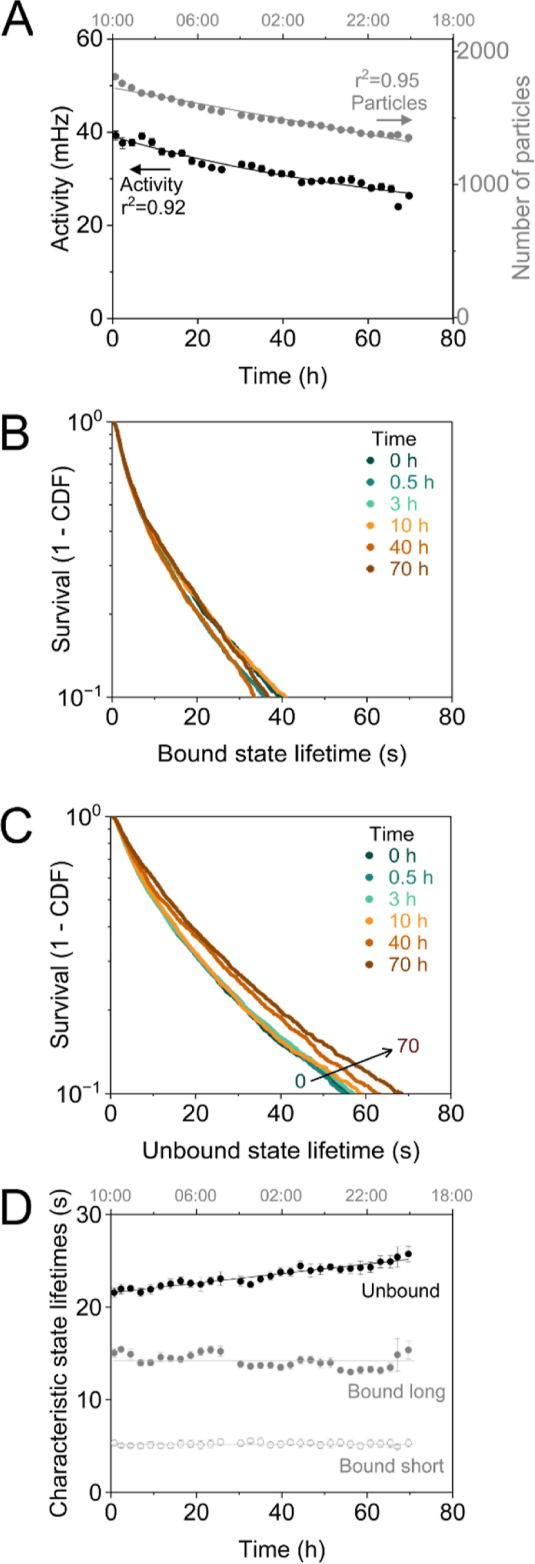
Time dependencies of activity signal, number of particles, and state lifetimes of a cortisol t-BPM sensor, without analyte and without flow. Tethered particles were coupled to the top inner surface of the flow cell; see the sketch in Figure 1A. (A) Switching activity (black) and the number of tracked particles (gray) for 71 h (about 3 days) of continuous measurements. A linear fit gives a relative loss rate of the switching activity of 0.44 ± 0.02 % per hour. (B) Distribution of bound state lifetimes plotted as survival curves (1-CDF, with CDF being the cumulative distribution function), measured after different aging times. The lifetime curves were fitted with a double-exponential decay function. The long characteristic lifetime (about 15 s) is attributed to the dissociation of single antibody–analogue bonds.^23^ (C) Survival curve of unbound state lifetimes, fitted with a multiexponential fit due to the heterogeneous distribution of binders on the particles.^26,27^ The unbound state lifetimes relate to the association process between particles and surface, which becomes less effective over time. (D) Characteristic bound and unbound state lifetimes plotted as a function of time. The characteristic unbound state lifetime increases over time. The bound-state lifetimes (both short and long) are constant over time. Lines are guides for the eye.

**Figure 4:**
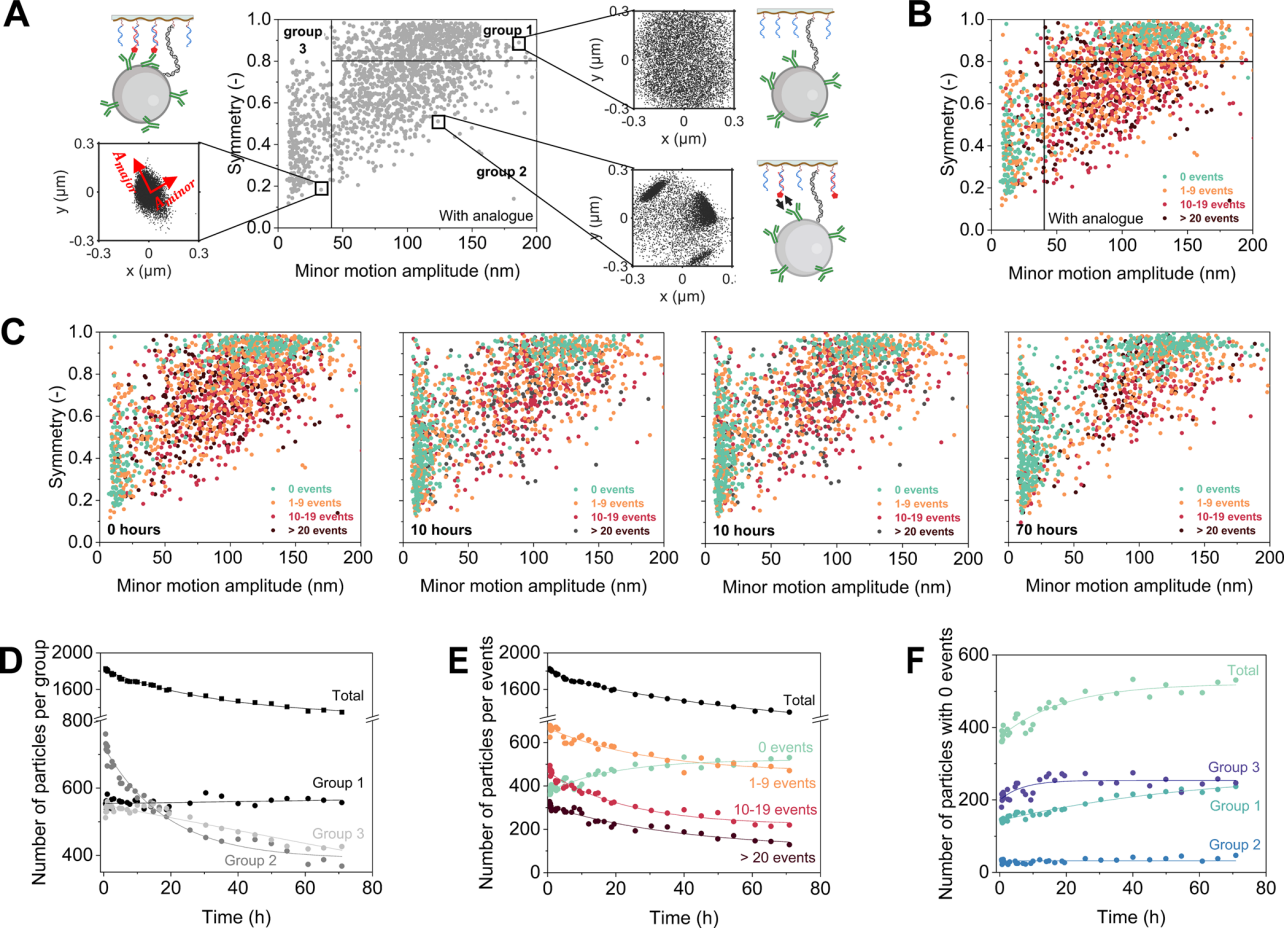
Distributions of motion patterns and switching behavior studied for 71 h for a t-BPM cortisol sensor in a flow cell without analyte molecules in solution and without flow. (A) Explanation of the concept of a particle motion distribution plot. In the plot, every individual particle of a BPM sensor is represented by a single point, expressing the symmetry and the minor amplitude of the particle’s motion pattern.^28^ The plot is separated into three groups. In group 1, the particles have disk-shaped motion patterns; these relate to single-tethered particles that are mostly in the unbound state. Particles in group 3 show very restricted motion, e.g., due to multivalent and/or nonspecific interactions. In group 2, the particles have intermediate behaviors and switch between unbound and bound states. (B) Particle motion distribution plot with dot colors to indicate the number of switching events of the individual particles: green represents nonswitching particles (0 events), orange represents low switching particles (1–9 events), red represents medium switching particles (10–19 events), and brown represents high switching particles (above 20 events), all corresponding to the same sensor as in panel (A). (C) Particle motion distribution plot as a function of time, for the same sensor as in panels (A) and (B), observed after 0, 10, 40, and 70 h. Before the experiment, the sensor surface was prepared with the analyte analogue, and the excess analogue was washed away with buffer. (D) Number of particles classified into three different groups based on their motion patterns, plotted as a function of time, derived from the data in panel (C). (E) Number of particles classified in terms of the number of switching events per particle (0, 1–9, 10–19, or more than 20 events), plotted as a function of time, derived from the data in panel (C). (F) Number of particles showing no switching events, classified in terms of their motion patterns (group 1, group 2, and group 3), plotted as a function of time, derived from the data in panel (C). Lines are guides for the eye.

The results of Figure 1B raise the question of which mechanisms may cause long-term changes in a competitive BPM biosensor. To answer this question, we focused on experiments under static conditions (without continuous exchange of fluid) and measurements over very long timespans (several days), as shown in Figure 1C. Here, a sensor was prepared with antibody molecules on the particles and analogue molecules on the sensing surface, the flow cell was flushed using a buffer solution without any analyte, and then the signal was recorded in static mode, i.e., without any flow of fluid. The switching activity was recorded for 78 h. The activity signal shows a gradual decrease as a function of time, with some fluctuations. A linear fit gives a relative signal loss rate of 0.40 ± 0.04 % per hour (see the fitted line), which means that 50% of the initial signal is lost after 3–4 days. The time-dependent signal undulates around the fitted line, showing fluctuations on a time scale of 12 or 24 h; we think this might be due to diurnal temperature fluctuations in the laboratory, since affinity-based interactions are sensitive to temperature. The observed gradual decrease can have several origins (see Figure 1D), such as changes in specific and/or nonspecific interactions between the particle and the surface. The changes may be caused by molecules somehow losing their functionality (e.g., antibodies, analogue molecules, blocking molecules), or the changes may be due to the detachment and subsequent release of molecules into the solution. To shed light on these mechanisms, we designed experiments with single-sided aging (Figure 2), with state-lifetime analysis (Figure 3), and with motion pattern analysis (Figure 4).

### Single-Sided Aging and Equilibrium-Shift Experiments

Figure 2 sketches an experiment that aims to separate long-term changes that occur due to the particles from those that occur due to the sensing surface. Figure 2A focuses on changes caused by the particles revealed in a particle-aging experiment. Batches of particles were prepared at different times and stored in a buffer solution at room temperature (RT). Subsequently, these particle batches, each having undergone a different aging duration, were evaluated on the same day, on freshly prepared sensing surfaces. Biofunctionalized slides were provided with six microfluidic measurement chambers, so that six particle batches could be simultaneously tested on a single slide. Each aged particle solution was supplied into an individual microfluidic sensing chamber to allow their interaction with the substrate.

The binding of each aged particle batch to the sensing surface was quantified in an f-BPM experiment,^19^ i.e., free biosensing by particle motion, without using a tether molecule between the particle and the surface (see Supporting Information SI2).^19^ The particles and surface were functionalized with specific amounts of antibody on the particles and analogue on the surface to obtain a bound fraction of about 70% as a starting point (see Figure S3), as this allows observations of increases as well as decreases in the bound fraction in an aging experiment. The f-BPM experiment was reproducible in different flow cells, particle batches, and experiment days (Figure S3 panels C–E), allowing comparisons of experimental results between different sensing surfaces, particle batches, and days.

Figure 2A top panel summarizes the particle aging experiment. Each batch of particles was prepared using the same experimental procedure. After functionalization, particles were diluted 2500 times in 0.5 M NaCl in PBS to 0.004 mg/mL and thereafter placed on a rotatory fin at RT for different aging times (0–170 h). The 0.004 mg/mL particle concentration is similar to the concentration in the long-term t-BPM experiment in Figure 1C. After the aging procedure, the aged particles were evaluated with both direct and competition assay f-BPM readout. In a direct assay, particles are added to the sensing surface without any analyte in solution, which reveals changes of specific as well as nonspecific binding between particles and the surface. In a competition assay, the particles are premixed with a high concentration of cortisol (30 μM) and then added to the sensing surface. The added cortisol blocks the specific binding sites on the antibodies, so the experiment reveals changes in nonspecific interactions between particles and the sensing surface.

The results in Figure 2A show that the bound fraction values as a function of aging time are constant with direct-assay readout and increase with competition-assay readout. This suggests that the nonspecific interactions between particles and surface increase over time, while simultaneously the specific interactions are reduced. The decrease of specific interactions can indicate that antibodies gradually lose their binding functionality and/or that antibodies are released from the particles.

The results in Figure S5A,B show that higher particle concentrations during aging experiments give a lower loss of particle functionality as neither the direct assay nor the competition assay readouts show significant changes as a function of aging time. We attribute the improved stability at high particle concentrations to the occurrence of antibody rebinding. For antibody rebinding to be effective, the concentration of dissociated antibodies needs to be higher than the *K*_d_ of the interaction between biotinylated antibodies and streptavidin-functionalized particles. Immobilized streptavidin and conjugated biotin have a dissociation rate constant on the order of *k*_off_ ∼ 10^–6^–10^–5^ s^–1^;^25^ so with a typical biomolecular association rate constant on the order of *k*_on_ ∼ 10^5^–10^6^ M^–1^ s^–1^, the estimated equilibrium dissociation constant (*K*_d_ = *k*_off_/*k*_on_) is in the range of 1–100 pM. In the 2500× dilution experiment, an assumed dissociation of 1% of the antibodies from the particles would lead to a concentration of antibody in solution on the order of about 1 pM. This concentration is smaller than the estimated *K*_d_, and thus antibody rebinding would not be effective in the 2500× dilution experiment. However, in the experiments with lower particle dilutions, the concentration of antibodies in solution would be higher than the *K*_d_ and, therefore, the rebinding of antibodies to the particles is expected to be more effective in those conditions. Thus, antibody rebinding may explain the lower loss of particle functionality observed in the aging experiments with high particle concentrations.

A loss of antibodies not only induces a reduction of specific interactions but can also lead to an increase of nonspecific interactions, since streptavidin becomes exposed and may subsequently interact with the sensing surface. In addition, a loss of blocking molecules (biotin-PEG and/or biotin-polyT) from the particles may also contribute to an increase of the nonspecific interactions between particles and the sensing surface. We hypothesize that these loss mechanisms may cause an increase of the bound fraction over time, as seen in the competition assay readout in Figure 2A.

An aging experiment of the sensing surface is shown in Figure 2B. Sensing surfaces were prepared by hybridizing analogue molecules for 60 min followed by a washing step with 0.5 M NaCl in PBS to remove any unbound analogue molecules. Surfaces were prepared at different time points and left to age over different durations (0–170 h). The aging was performed at RT in a humidity chamber to prevent fluid evaporation. After the aging process, the sensing surfaces were evaluated by performing an f-BPM experiment with freshly prepared particles, as illustrated in Figure 2B. The aged surfaces were evaluated with direct-assay readout (no cortisol) and competition-assay readout (high cortisol concentration). The competition-assay readout shows a bound fraction that remains low, without any dependence on the aging time, so no increases of nonspecific interactions are observed. However, the direct-assay readout shows a strong decrease of the bound fraction as a function of aging time. This points to a loss of specific binding functionality of the sensing surface, possibly due to analogue molecules gradually losing their binding functionality or analogue molecules being released from the sensing surface. A release could be due to dehybridization of the ssDNA–ssDNA bond, or due to dissociation of PLL-*g*-PEG molecules from the substrate.

To clarify the underlying origin, an equilibrium-shift experiment was performed, where sensing surfaces were aged with or without the presence of analogue molecules in the solution. If analogue functionality loss is the dominant mechanism, then the aging effects should be independent of the presence of analogue molecules in solution. However, if analogue dissociation is the dominant mechanism, then aging effects should be smaller in the presence of analogue in solution, as this would shift the equilibrium toward analogue molecules coupled to the sensing surface. Figure S5C shows the results for sensing surfaces aged in the presence of analogue molecules in solution for direct- and competition-assay readouts. In both cases, no changes in the bound fraction are seen as a function of aging time. This implies that the decrease in bound fraction in Figure 2B was mainly due to the dissociation of analogue molecules instead of a loss of functionality of analogue molecules coupled to the sensing surface.

In summary, the single-sided aging and equilibrium-shift experiments of Figures 2 and S5, evaluated with f-BPM readout, show that changes of the particles and the sensing surface occur on time scales of tens of hours and that the changes are caused by dissociations of binder molecules. The dissociation of molecules was proven in the equilibrium-shift experiments: (i) by demonstrating that long-term changes are reduced in the case of higher surface-to-volume ratios during the particle aging process, tuned by varying the concentration of particles in solution and (ii) by demonstrating that long-term changes are reduced in the presence of analogue molecules in solution during the surface aging process.

### Switching Activity and State Lifetimes

In the t-BPM sensor, switching events of particles are observed due to single-molecular interactions. The time characteristics of the switching events reflect the effective bound and unbound state lifetimes of the particles (see Figure S1). Figure 3 investigates how state lifetimes change during long-term BPM experiments. Figure 3A shows the results for a cortisol t-BPM sensor without any flow (as shown in Figure 1C). Panel A indicates that the sensor shows a gradual reduction of the switching activity (black points, left *y*-axis, decrease at a rate of 0.44 ± 0.02 % per hour) and of the number of tracked particles (gray points, right *y*-axis, decrease at a rate of 0.39 ± 0.01 % per hour).

The number of tracked particles decreases over time because when a tether molecule dissociates or breaks, the corresponding particle sediments away from the sensing surface, as the sensor cartridges were operated with particles attached to the top surface of the microfluidic flow cell (Figure 1A). Interestingly, the particle loss rate depends on the functionalization of particles and surface, as the loss rate appears to be larger for control measurements without specific interactions (Figure S6). We attribute this observation to a larger retethering probability of particles that undergo specific antibody–analogue interactions. The specific interactions reduce the time-averaged distance between particles and the sensing surface, enhancing the probability that a dissociated tether molecule can reattach to the particle.

Figure 3B,C shows how the distributions of bound and unbound state lifetimes of the switching particles depend on the aging time. Figure 3D summarizes the observed state lifetimes, where the characteristic bound-state lifetimes were determined by double-exponential fits (short bound-state lifetimes due to nonspecific bonds and signal processing artifacts; long bound-state lifetimes due to antibody–analogue bonds),^23,26^ and the characteristic unbound-state lifetimes were determined by multiexponential fits (related to log-normal distributed mean unbound state lifetimes).^23,26^ The data show that the bound-state lifetimes do not depend on the aging time, indicating that the nature of the bound states of the particles is unaltered, in agreement with the expectation that the particle bound states are dominated by single-molecule interactions between anticortisol antibodies and the cortisol analogue. In contrast, the unbound-state lifetimes shift toward longer lifetimes as a function of aging time. This is in agreement with the hypothesis that binder molecules gradually dissociate from the particles and the sensing surface.

### Distributions of Particle Motion and Switching Activities

BPM is based on the tracking of hundreds to thousands of individual particles on a sensing surface. Every particle functions as a sensor and contributes to the total signal. It is advantageous to use a high number of particles, as this improves the statistics and the analytical performance.^22^ However, individual particles can have different properties. For example, some particles may show a higher switching rate compared to other particles, which may be related to the number of antibody and analogue molecules that are accessible in the region between the particle and the sensing surface. Figure 4 analyzes the differences between particles by studying their motion patterns. The motion pattern of a particle is a cumulative scatter plot of particle center positions recorded over a given period of time (5 min in this experiment). The minimum amplitude of a motion pattern is determined by the resolution of the imaging system and the data analysis; it is around 10 nm for the setup used in this paper. The maximum amplitude is given by the maximum in-plane displacement of a particle with a stretched tether, which is around 200 nm for the t-BPM sensor of this paper.

Motion patterns of particles can differ strongly, depending on how the particles interact with the sensing surface.^28^ For example, disk-like motion patterns are seen for particles that are coupled to the surface by a single tether molecule and that do not form other bonds with the surface. On the other hand, when a particle is stuck due to multivalent bonds or strong nonspecific interactions, then the particle shows very little motion, and the motion pattern is strongly restricted.

Figure 4A shows a distribution plot that represents all particles of a t-BPM sensor by positioning the particles according to the properties of their motion patterns. The motion pattern of every particle was analyzed by the motion amplitudes along their major (*A*_major_) and minor (*A*_minor_) motion axes, calculated from the covariance matrix of the position data.^17^ These parameters were then used to represent the particles in the distribution plot, with the minor motion amplitude on the *x*-axis and the symmetry of the motion pattern (*A*_minor_/*A*_major_) on the *y*-axis, the latter ranging between 0 and 1. In this distribution graph, three groups are defined that represent particles with different motion behaviors. Group 1 contains particles that have motion patterns with high rotational symmetry (symmetry >0.8) and significant motion (minor motion amplitude >30 nm). This group contains disk-like motion patterns that are characteristic for single-tethered particles that do not form other bonds with the surface. Group 3 contains particles with very little motion (minor motion amplitude <30 nm), characteristic for particles that are stuck. Group 2 contains particles with significant motion (minor motion amplitude >30 nm) and motion patterns that deviate from rotational symmetry (symmetry <0.8). This group contains particles that are tethered and have heterogeneous motion patterns due to additional binding spots.

Figure 4B adds another layer of information by expressing the switching activity of every individual particle with a color classification. The data show that nearly all particles in group 2 have a nonzero switching activity (very few points are green with 0 switching events), while many particles in groups 1 and 3 do not show switching activity (most points are green). Figure 4C depicts how the motion distribution plot changes as a function of aging time (0, 10, 40, and 70 h). The data show clear changes over time, particularly for the particles in group 2. The time dependencies of the number of particles in different groups are quantified in Figure 4D. At the starting condition, this sensor contains particles mostly in group 2, but after 70 h group 2 has decreased by 50% and group 3 has decreased by 20%, resulting in a sensor where most particles are in group 1. Figure 4E bins the particles according to their switching events, showing that the number of switching particles decreases and the number of nonswitching particles increases over time. Finally, Figure 4F shows that the increase of nonswitching particles (green dots) is caused by an initial increase in group 3 and a gradual increase in group 1. At all times, group 2 has a very low number of particles without switching events. The increase in group 3 (representing the stuck particles) can be attributed to particles that are initially in group 2 (switching particles) and over time migrate to group 3 (they become nonswitching particles), due to nonspecific and multivalent interactions. The largest change is visible in group 1, attributed to the migration of particles from group 2 to group 1 due to the loss of binder molecules (antibodies and analogues). Further support for these hypotheses is given in Supporting Information 7 and 8 (motion maps of tethered particles before activation and particle distribution over time for t-BPM negative controls).

The leading hypotheses of Figure 2, resulting from the single-sided aging and equilibrium-shift experiments with f-BPM readout, are that (i) the surface aging results in a loss of analogue molecules and that (ii) the particle aging causes the dissociation of antibodies and an increase in nonspecific interactions. The lifetime study of Figure 3 shows that the unbound state lifetimes increase, which can be attributed to a gradual loss of binder molecules on particles and/or the sensing surface. These hypotheses are in agreement with the t-BPM aging experiment in Figure 4 because the data show that switching particles become nonswitching particles via two different pathways: particles migrate from group 2 to group 3 (due to nonspecific and/or multivalent interactions) and particles migrate from group 2 to group 1 (due to losses of binder molecules), with the latter dominating on time scales of tens of hours. Thus, the unifying conclusion of the experiments is that the long-term changes observed in the BPM cortisol sensor are not caused by functionality losses of antibodies or analogue molecules, e.g., due to denaturing or due to nonspecific sticking of the molecules, but rather by the release of the molecules from the particles and the sensing surface.

## Conclusions

The research of this paper has addressed the question as to which molecular processes drive the changes observed over several tens of hours in a biosensor based on particle motion, where biofunctionalized particles interact with a biofunctionalized sensing surface. Experimental methodologies were developed for studying single-sided aging in various conditions (Figure 2), for quantifying long-term changes in bound and unbound state lifetimes (Figure 3), and for studying long-term changes in distribution plots of motion pattern properties and switching activities (Figure 4). These methodologies were applied to a cortisol BPM sensor with antibodies on the particles and cortisol analogue on the sensing surface, under conditions without fluid flow. The results have revealed that long-term changes are dominantly caused by gradual losses of antibodies from the particles and analogue molecules from the sensing surface, both occurring at loss rates of a few tenths of a percent per hour. In addition, particle aging causes an increase of nonspecific interaction between particles and the sensing surface, due to antibody release from the particles and/or loss of blocking molecules.

Noncovalent coupling methods are very practical for biosensor fabrication, as these are fast and easy to control. The studied cortisol sensor contains several noncovalent couplings. The biotin–streptavidin interaction was used to couple molecules to the particles: it was used for coupling biotinylated antibodies to the particles, for coupling blocking molecules to the particles (involving biotin-polyT and biotin-PEG), and for coupling tether molecules to the particles. On the sensing surface, electrostatic interactions were used to couple PLL-*g*-PEG to the polymer substrate, and cortisol–ssDNA conjugates were coupled to ssDNA-functionalized PLL-*g*-PEG molecules using 20 base pair hybridization. The results of the experiments in this paper indicate that the long-term changes in the biosensor are dominantly caused by the dissociation of biotin–streptavidin and ssDNA–ssDNA bonds, with release rates on the order of a few tenths of a percent per hour. The data show that the resulting release rates become limiting for continuous sensor operation over long spans of time. In further research, we will study alternative sensor designs with different coupling strategies and quantify their long-term changes using the methods developed in this paper.

It is important to develop diverse research methodologies to quantify long-term changes and unravel their molecular origins because long-term operation is an important aim for continuous biosensors.^10,14,16,29−31^ The methodologies in this article were studied on a competition-based small-molecule BPM sensor. We think that the methodologies can also be applied to other affinity-based sensors, especially the single-sided aging experiments and the equilibrium-shift experiments.^6,10−16,18^ The experiments of this paper were performed using buffer solutions; as a next step, it will be relevant to study how long-term changes are affected by the composition of the fluid. Also, transport properties need to be considered; analyte transport in a continuous sensor is driven by advection and/or diffusion,^32^ so it is relevant to study how these mechanisms affect the long-term changes of the biosensor. Furthermore, tracking individual particles over the whole measurement period, together with modeling and simulations, can provide further insights into the molecular origins of signal changes. Finally, thermally activated inter- and intramolecular processes are expected to play important roles, so it will be interesting to study long-term changes as a function of temperature, for gaining scientific insights, as well as for developing accelerated aging protocols that can be used to predict long-term sensor changes based on experiments with short durations. We foresee that this is a rich area of research that will enable the development of biosensors that can operate continuously over days and weeks.

## Materials and Methods

### Preparation of a Tethered-BPM Cortisol Sensor

Cartridges with premade flow channels (SL003010, ibidi GmbH) were cleaned with Milli-Q water in a sonication bath (Branson 2800) for 10 min. The cartridges were dried under a nitrogen stream and placed in a UV ozone cleaner (Digital UV Ozone System, Novascan) for 30 min. Afterward, the cartridges were sealed with optically transparent tape (no. 232702 Sealing tape, Thermo Scientific). The preparation of the cartridge was done with a manual pipette. 50 μL of polymer solution with 0.45 mg/mL poly(l-lysine)-grafted poly(ethylene glycol) (PLL-*g*-PEG, SuSoS) and 0.05 mg/mL azide-functionalized PLL-*g*-PEG (azide-PLL-*g*-PEG, Nanosoft Biotechnology LLC) in Milli-Q water was added into the flow channel of the cartridge. The cartridge was incubated for 3 h in a humidity chamber to allow physisorption of the polymer onto the flow channel. After that, the polymer solution was removed, and 50 μL solution with 0.5 nM DBCO-dsDNA-biotin tether molecules (221 bp dsDNA)^23^ was added and incubated overnight. Finally, the tether solution was replaced by adding 50 μL of solution containing 2 μM DBCO-ssDNA capture molecules (DBCO—5′—GTG CGG CAG GGG TAA GAC CA—3′) in 0.5 M NaCl in PBS, incubated in a humidity chamber for at least 72 h, followed by a washing step with 0.5 M NaCl in PBS to remove the unbound capture molecules.

For functionalizing the particles, 4 μL solution with 250 nM biotinylated anticortisol antibody (prepared as described in Van Smeden’s publication^23^) was mixed with 4 μL of 10 mg/mL streptavidin-coated magnetic particles (Dynabeads MyOne Streptavidin C1, 65001, Thermo Scientific) and incubated for 30 min at RT in a rotatory fin. A 3 μL solution with 10 μM biotinylated polyT (biotin- 5′- TTT TTT TTT TTT TTT T - 3′) and 12 μL of PBS were added to the 8 μL particles mixture and incubated for 45 min to partially block the streptavidin molecules. After incubation, functionalized particles were washed with PBS containing 0.05% Tween-20 (PBST) and resuspended in 600 μL of 0.5 M NaCl in a PBS. Lastly, the suspended particles were sonicated in a sonication bath (Branson 2800) for 30 s to disaggregate particle clusters before use.

The flow cell was connected to a 10 mL syringe (Norm Jet) on one end and to a microfluidic rotary valve (LSPone, Advanced Microfluidics) on the other end using luer locks (no. 10000081, ChipShop) and flexible silicone tubing (no. 45630104, Freudenberg Medical). The flow cell was placed on a homemade compact microscope setup. 200 μL of particles functionalized with anticortisol antibodies was flushed through the functionalized ibidi cartridge (Harvard pump 11 Elite, 100 μL/min withdrawal speed). Subsequently, the cartridge was flipped to allow particles to sediment toward the sensing surface and become tethered during 15 min. Afterward, 200 μL of solution with 100 μM 1 kDa mPEG-biotin (PG1-BN-1k, Nanocs; blocking solution) was flushed through the flow cell and incubated for 10 min, in order to block the remaining free streptavidin molecules on the particles. Thereafter, the cartridge was flipped, in order to operate and study the sensor with particles attached to the top surface of the microfluidic flow cell (see Figure 1A). To activate the sensor, 200 μL with 700 pM of cortisol–ssDNA conjugate (prepared as described in Van Smeden’s publication^23^) was added to the flow cell and incubated for around 20 min. During the functionalization with analogue molecules, the activity signal was monitored in order to reach a signal of about 35 mHz. Afterward, 200 μL of solution with 0.5 M NaCl in PBS was flushed through the flow cell to remove any unbound analogue molecules.

For measuring the cortisol dose–response curves, cortisol solutions in the concentration range of 0–30 μM were transported into the flow cell channel at a flow rate of 100 μL/min over a duration of 2 min. Subsequently, the motion of particles was recorded over 5 consecutive intervals of 1 min each in the absence of flow.

For the long-term measurement of the t-BPM cortisol sensor without flow, the t-BPM signal was recorded every hour over a total period of about 80 h. The measurements were conducted in PBS containing 0.5 M NaCl, without cortisol in solution, and in the absence of flow.

### Aging of Particles and Sensing Surface with f-BPM Readout

Polymer slides without premade flow channels (SL000001-V, ibidi GmbH) were used for the f-BPM experiments. These slides were made of the same polymer material as the ibidi cartridges in the t-BPM experiments. The slides were sonicated in Milli-Q water for 10 min, then dried with a nitrogen stream, and put in a UV ozone cleaner (Digital UV Ozone System, Novascan) for 30 min. Custom-made flow-cell stickers with six 25 μL chamber volumes (Grace Biolabs) were placed on top of the ibidi slides. Fluids were supplied into the chambers using manual pipettes. 25 μL of solution containing 0.45 mg/mL of PLL-*g*-PEG and 0.05 mg/mL azide-PLL-*g*-PEG was added to the flow cells. The slides were incubated for 3 h in a humidity chamber to allow physisorption of the polymer onto the surface. Thereafter, the polymer solution was replaced by 25 μL of solution with 2 μM DBCO-ssDNA capture molecules in 0.5 M NaCl in PBS. The solution was incubated for at least 72 h and then washed with PBS containing 0.5 M NaCl to remove the unreacted capture molecules. Finally, 25 μL of cortisol-ssDNA (analogue) solution in PBS containing 0.5 M NaCl was added to the flow cell and incubated for 60 min before adding the particles for measurement.

Particles were functionalized in a similar way as in the t-BPM experiment, with 4 μL of particles mixed with 4 μL of biotinylated cortisol antibody and incubated for 30 min at RT. Then, 10 μL of solution with 10 μM biotinylated polyT was added to the particle solution to block the streptavidin molecules. The functionalized particles were washed in PBST and resuspended in 600 μL of 0.5 M NaCl in PBS and subsequently sonicated in a sonication bath for 30 s to disaggregate particle clusters before storage. To achieve the starting bound fraction of 0.7, titrations of antibody concentration on the particles (0, 62.5, 125, 250, 500, and 1000 nM) and analogue concentration on the sensing surface (0, 125, 250, 500, 1000, and 2000 pM) were performed. The reproducibility of the sensor preparation was evaluated by employing 12 flow cells, four batches of particles, and two consecutive measurement days. For each flow cell, three different spots were chosen to show the homogeneity of the flow cell. 250 nM antibody concentration and 700 pM analogue were used in the aging experiments.

In the free-BPM experiments, the particles were added to the flow cell with a manual pipette. For direct assay readout, the particle suspensions were added to the functionalized flow cells for 30 min for particle sedimentation, and images of particles were recorded to get the bound fraction of the particles. For competition assay readout, particle suspensions were first incubated with 30 μM of cortisol for 30 min and then added to the prepared sensing surface and measured after 30 min of sedimentation.

For particle aging, functionalized particles were prepared on different days and were suspended in different volumes of PBS containing 0.5 M NaCl (0.004, 0.07, and 0.8 mg/mL particle concentration) and placed in a rotatory fin at RT for 1 day and up to a week. After a week, particles with different aging times and freshly prepared particle suspensions were all further diluted to 0.004 mg/mL particle concentration and added to freshly prepared sensing surfaces. The bound fraction was measured with both direct and competition assay readout. For the aging of sensing surfaces, analogue molecules were added to the sensing surfaces, incubated for 60 min, kept as is, or replaced with PBS containing 0.5 M NaCl for aging. The aging of sensing surfaces in PBS containing 0.5 M NaCl ranged from 1 day to a week. After 1 week, freshly prepared particles were added to the aged and freshly prepared sensing surfaces. The bound fraction was measured with both direct and competition assay readout.

### Particle Imaging and Data Analysis

In t-BPM experiments, particles were imaged by bright-field microscopy on a homemade compact microscope setup with 10× magnification and a green LED light source. A high-speed camera (FLIR GS3-U3-32S4M-C) was used with a field of view of 1.28 × 0.96 mm^2^. The images were acquired at a framerate of 30 Hz and an exposure time of 0.5 ms. A maximum-likelihood multiple-windows change point detection algorithm (MM-CPD)^24^ was employed to analyze the motion of tethered particles in terms of activity, lifetimes, and motion patterns. The minor motion amplitude and symmetry were employed to establish motion pattern distribution maps of the tethered particles.

In f-BPM experiments, particles were imaged by bright-field microscopy using a homemade compact microscope setup with 10× magnification, a green LED light source, and a motorized *XY* stage (ASR series 100 × 120 mm travel, Zaber Technologies Inc.). A high-speed camera (FLIR Blackfly S BFS-U3-31S4M) was used with a field of view 0.53 × 0.71 mm^2^. Particle motion was recorded for 0.5–1 min blocks with a frame rate of 60 Hz and 3 ms exposure time. The recorded frames from each measurement were analyzed in real-time using a particle tracking software described by Bergkamp et al.^22^ Diffusivity time traces were derived from the tracked particle data, with the bound fraction as the output parameter obtained from the diffusivity traces, as detailed in Supporting Information SI2.

### Definitions

The following definitions were used, as proposed in ref (22): *Continuous monitoring* refers to a process and technology to continuously collect measurement data from a system of interest. *Continuous biosensing* refers to continuous monitoring using a biosensor. *Real-time continuous biosensing*, or *real-time biosensing* in short, refers to continuous biosensing with a time delay that is small with respect to the time scales of typical fluctuations in the system of interest.

## Supporting Information Available

BPM assay optimization, BPM assay reproducibility, equilibrium-shift assay conditions, BPM state lifetimes, number of particles over time, motion distribution maps, and particle distribution over long-time spans (PDF)

## Abbreviations

BPM: biosensing by particle motion
CRT: cortisol
MM-CPD: maximum-likelihood multiple-windows change point detection
CDF: cumulative distribution function
DRC: dose–response curve
