# Supplementary material for: Molecular Origins of Long-Term Changes in a Competitive Continuous Biosensor with Single-Molecule Resolution
Source: ACS Sens. 2024 Jul 5;9(7):3520–30. doi: 10.1021/acssensors.4c00107 (PMC11287755; doi:10.1021/acssensors.4c00107)
Supplement: Supplementary file 1 — se4c00107_si_001.pdf [file se4c00107_si_001.pdf]

Molecular origins of long-term changes in a competitive continuous biosensor with single-molecule resolution

Sebastian Cajigas[a,b], Arthur M. de Jong[c,d], Junhong Yan[a], Menno W.J. Prins[a,b,c,d*]

[a] Helia Biomonitoring, 5612 AR, Eindhoven, The Netherlands
[b] Department of Biomedical Engineering, Eindhoven University of Technology, 5612 AZ, Eindhoven, The Netherlands
[c] Department of Applied Physics, Eindhoven University of Technology, 5612 AZ, Eindhoven, The Netherlands
[d] Institute for Complex Molecular Systems (ICMS), Eindhoven University of Technology, 5612 AZ, Eindhoven, The Netherlands
*Corresponding author: m.w.j.prins@tue.nl

Supplementary Information

Table of Contents

SI 1: State lifetimes in biosensing by particle motion 2
SI 2: Basic principle of free BPM 2
SI 3: Antibody and analogue titrations and study of reproducibility 5
SI 4: Duplicate particle and surface aging experiments 6
SI 5: Aging experiments with equilibrium-shift conditions 7
SI 6: Number of tethered particles in the BPM sensor over long time spans 9
SI 7: Tethered particles before analogue addition 10
SI 8: Particle distributions over time for t-BPM negative controls 11
References 12

SI 1: State lifetimes in tethered biosensing by particle motion

In tethered Biosensing by Particle Motion (t-BPM), hundreds to thousands of particles are attached to a sensing surface via a flexible DNA tether molecule[1–6]. Through specific binders on both particles and the sensing surface, transient binding events occur, giving bound and unbound states. The bound state is characterized by confined particle motion while the unbound state shows a more extended motion pattern[7], as depicted in Figure S1 left panel.

In t-BPM, the particle positions are recorded by video microscopy and the particles are tracked by a localization algorithm[1,8]. A change point detection algorithm identifies the (un)binding events between particles and the sensing surface, arising from the intermittent interactions between antibodies and analogue molecules.

Figure S1 right panel illustrates the xy-positions of a single particle tracked over 300 seconds. Vertical green lines denote instances where changes in particle motion occur. The time intervals between consecutive switching events correspond to state lifetimes of the particles.

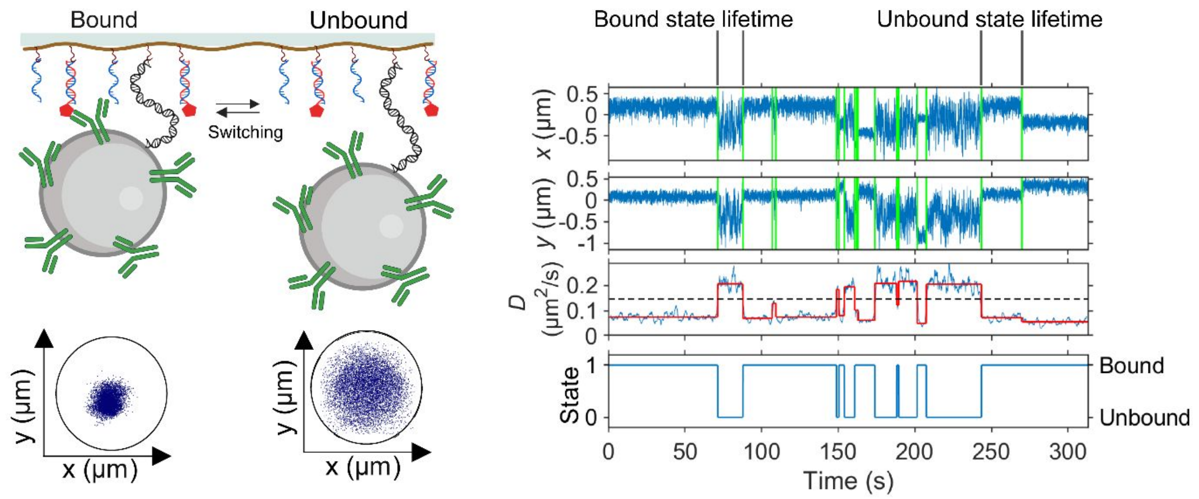

Figure S1. In t-BPM, particles switch between bound and unbound states (left) due to the specific interactions between antibody and analogue molecules. The x and y positions of the particles over time are shown in the right graph, together with the diffusion coefficient of the particle calculated from the x and y positions. The bound and unbound states of the particle can be determined from the x, y positions and the diffusion coefficients. The time span between two consecutive events is defined as the state lifetime.

SI 2: Basic principle of free BPM

This paper studies long-term changes in a cortisol t-BPM sensor (i.e. tethered-BPM sensor) with a competitive design, as sketched in Fig. 1. To clarify the molecular origins of long-term changes, experiments with separate aging of particles and surfaces were performed, with readout using biosensing by particle motion using free particles (i.e. free-BPM sensor), see Fig. 2. In this Section, the basic principles of f-BPM are explained.

Free Biosensing by Particle Motion (f-BPM) is based on the continuous tracking of the Brownian motion of functionalized particles suspended above a functionalized sensing surface. Figure S2A illustrates a competitive free BPM assay configuration where streptavidin-coated particles are functionalized with biotinylated antibodies (anti-cortisol antibody), and the sensing surface is coated with a low-fouling PLL-g-PEG bottle brush polymer layer that allows the covalent immobilization of DBCO-ssDNA capture molecules. In the presence of cortisol-analogue, these molecules hybridize to the ssDNA capture molecules on the sensing surface, enabling the formation of specific reversible bonds (switching) between antibodies on the particle and cortisol analogue on the sensing surface.

In the presence of high analyte concentration (cortisol) in solution, switching is interrupted because cortisol molecules occupy the binding sites on antibodies, thereby inducing particles to exhibit free Brownian motion without interacting with the sensing surface. This results in the particles remaining in the unbound state, characterized by a high diffusivity value, D ($\mu m^2/s$). The effective diffusivity is determined as a function of time from the mean squared displacement (MSD) of the particle[9]. In the absence of analyte (cortisol) in solution, particles show periods with reduced diffusivity due to temporal reversible interactions between particles and the sensing surface. The temporally reduced diffusivity indicates a transition of the particle into a bound state, accompanied by a significant decrease in the D value. Given the reversibility of the interactions between particles and the sensing surface, particles can dynamically switch between bound and unbound states. To distinguish between bound and unbound states, particle trajectories are constructed by tracking the center of the particles across all the recorded video frames, as illustrated in Figure S2B[9].

To distinguish between bound and unbound states, D values below a specific threshold are considered as bound states, while D values above the threshold are classified as unbound states. Figure S2B shows recorded particle trajectories and the attributed bound and unbound state traces as a function of analyte concentration. Figure S2B top panel depicts a particle trajectory, and D time trace for a particle at high concentration of cortisol. The D values for this particle, with analyte-occupied binding sites, are above the threshold ($D > 0.04\ \mu m^2/s$) throughout the measured time, indicating that the particle stays in the unbound state without undergoing any observed state transitions. Conversely, the bottom panel of Figure S2B displays a particle trajectory and D time trace for a particle at a low concentration of cortisol. In this case, temporal confinement of the particle (depicted by red dots) is observed along with the state transitions indicated by changes in the D value, ($D < 0.04\ \mu m^2/s$). The state transitions demonstrate the dynamic switching behavior of the particles between states.

The recorded D values are used to generate diffusivity histograms. The top panel of Figure S2C shows diffusivity histograms obtained from an entire population of tracked particles (~600) in a field-of-view at high concentration of cortisol. Similarly, the bottom panel of Figure S2C shows diffusivity histograms for the total population of tracked particles at low cortisol concentration. From the diffusivity histograms, the bound fraction, a characterization parameter for f-BPM, can be derived. The bound fraction is defined as the ratio of measured diffusivity values below a diffusivity threshold, indicating the population of bound states (N_{bound}) to the total number of diffusivity values ($N_{total} = N_{bound} + N_{unbound}$), which includes all tracked particles within the field of view for a given measurement frequency[10]:

$$\text{Bound fraction} = N_{\text{bound states}} / (N_{\text{bound states}} + N_{\text{unbound states}})$$

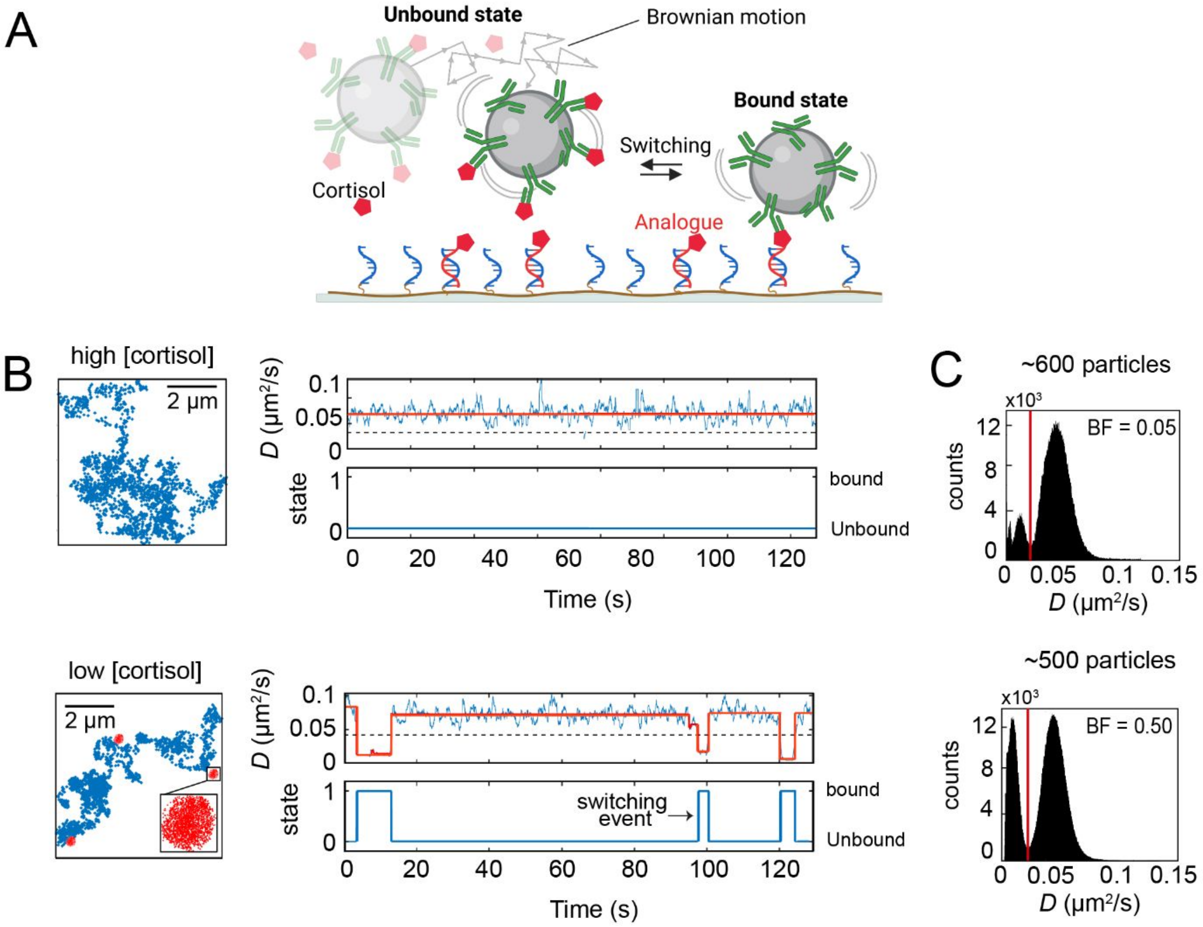

Figure S2. A) Biosensing by free particle motion (f-BPM) sensor with particles (Dynabeads MyOne) functionalized with biotinylated anti-cortisol antibodies, interacting with a sensing surface that is functionalized with cortisol-analogue molecules. The sensing surface consist of a polyethylene-derivative substrate, functionalized with a polymer layer (PLL-g-PEG/azide-PLL-g-PEG bottle brush polymer) to allow covalent immobilization by click chemistry of DBCO-ssDNA capture molecules. At this stage, particles exhibit free Brownian motion and are mainly in the unbound state. Subsequently, when cortisol-analogue is supplied (a cortisol-modified ssDNA) [11], these hybridize to the ssDNA capture molecules on the sensing surface. The presence of the analogue molecules enables the formation of specific reversible bonds between antibodies on the particles and cortisol-analogue on the sensing surface, allowing the observation of switching events between bound and unbound states. B) Experimental data for a competition cortisol f-BPM sensor. The top panel displays the trajectory of a particle at high concentration of cortisol. The bottom panel shows a trajectory of a particle at low cortisol concentration. The particle displays three confined spots attributed to its reversible interactions with the sensing surface. Switching particles dynamically transition between bound and unbound throughout a measurement period. C) In the top panel, the distribution of *D* for about 600 particles at high concentration of cortisol is shown, predominantly displaying unbound states. In contrast, the bottom panel shows the distribution of *D* for about 500 particles at low cortisol concentration, exhibiting both bound and unbound states.

SI 3: Antibody and analogue titrations and study of reproducibility

The aging experiments of Fig. 2 require a tuning of the antibody density on the particles and the analogue density on the sensing surface in order to have a starting condition with an f-BPM bound fraction signal of about 70%, as this allows observations of increases as well as decreases of bound fraction due to aging effects.

Figures S3A and B show two titration experiments to determine suitable concentrations of antibody and analogue in the preparation of an f-BPM sensor. Concentrations of 250 nM antibody and 500 pM of analogue were selected as the preparation conditions for particles and sensing surface, respectively.

Reproducibility experiments were carried out by employing different flow cells, particle batches, and different slides, see panels C-E. Under the different conditions tested (flow cells, particle batches, and slides) the experimental system showed to be highly reproducible in giving an average bound fraction of 70%. One-Way Analysis of Variance (ANOVA) showed that there were no significant differences among the mean values of particle batches and different slides tested.

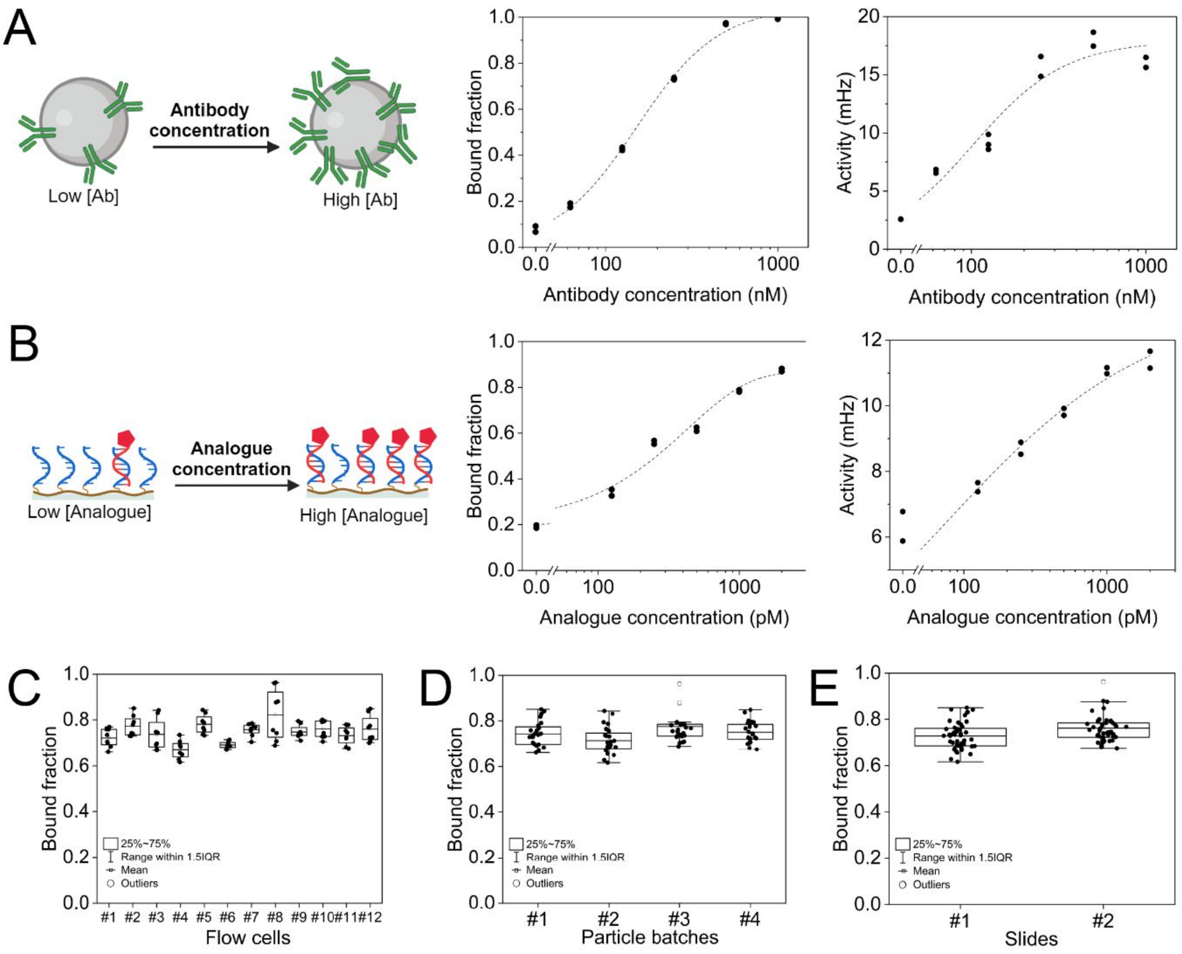

Figure S3. Titration of antibody and analogue concentration and reproducibility of free-BPM (f-BPM). A) Antibody titration in the range from 0 to 1000 nM, with 500 pM analogue on the sensing surface, with readouts of bound fraction and activity. B) Analogue titration from 0 to 2000 pM on the sensing surface, with 250 nM of antibody on the particles, with readouts of bound fraction and activity. C) Twelve flow cells were used to test the reproducibility of the f-BPM sensors. Within each flow cell, three different measurement spots (after inlets, centers, and before outlets) were selected to test the homogeneity of the flow cell functionalization. D) Four independently prepared batches of particles were measured in triplicate, on three separate flow cells. The variability within each particle batch was also investigated by measuring in three different spots along the flow cell. E) Two Ibidi microscope slides with six flow cells each, were measured on two consecutive days. The inter-day variability was investigated by measuring each flow cell at three different spots. The reproducibility of different particle batches and consecutive measurement days was analyzed with a One-way ANOVA test with a significance level of 0.05. One-way ANOVA test indicated that the mean values for the particle batches and the measurements days with p values, $p>0.74$ and $p>0.77$, respectively, are not significantly different. Dashed lines are guides to the eye.

SI 4: Duplicate particle and surface aging experiments

Figure 2 presented the single-sided aging of particles and sensing surfaces, with readout using an f-BPM sensor format. In this Section, duplicate measurements are reported for both particle and sensing surface aging, with both direct and competition assay f-BPM readout.

Figure S4A shows a duplicate particle aging experiment. Similar to the data in Figure 2A, the bound fraction values as a function of the aging time are constant for the direct assay readout. However, for competition assay readout, the bound fraction values display an increase with the aging time. Figure S4B shows a duplicate sensing surface aging experiment, in which the analogue solution was removed after 1 hour of incubation. As in Figure 2B, the bound fraction values of the direct assay readout decrease with the aging time, and the competition assay readout values remain constant over time.

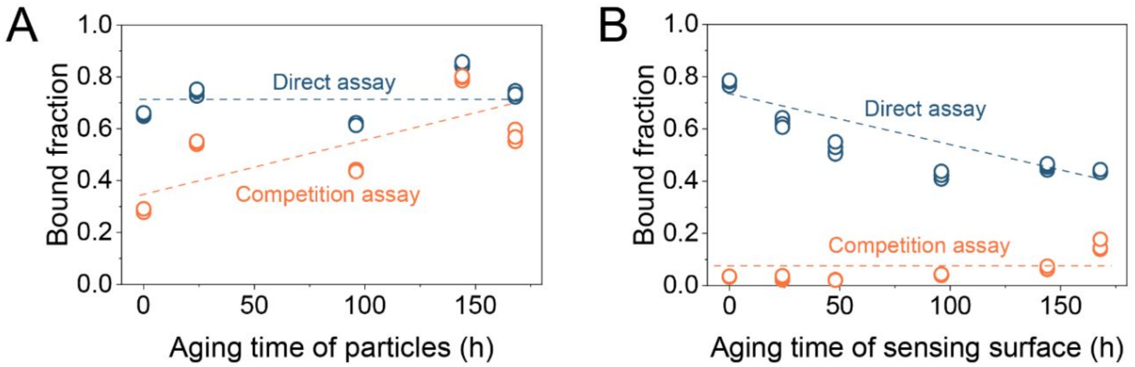

Figure S4. Duplicate aging study with f-BPM readout. Either particles or sensing surfaces were aged, with direct assay readout or competition assay readout using f-BPM. A) Particles with anti-cortisol antibodies were prepared on different days, distributed over a period of 7 days as described in the caption of Figure 2A. On the last day, differently aged particles were suspended in buffer without cortisol (direct assay, dark blue) or suspended in buffer with 30 µM of cortisol (competition assay, light blue) and then added to sensing surfaces freshly prepared with 500 pM analogue. B) Sensing surfaces with cortisol analogue were prepared by adding 500 pM analogue on different days, distributed over a period of 7 days. The analogue was incubated for 1 hour and thereafter the flow cells were washed with 0.5 M NaCl in PBS. The sensing surfaces were then stored in 0.5 M NaCl in PBS at room temperature in a humidity chamber to prevent drying of the fluidic channels during the aging time. On the last day, all prepared sensing surfaces were studied with freshly prepared particles, suspended in buffer without cortisol (direct assay) or suspended in buffer with 30 µM cortisol (competition assay). The bound fraction was measured in a BPM experiment with free particles (f-BPM). Dashed lines are guides to the eye.

SI 5: Aging experiments with equilibrium-shift conditions

Figure 2A shows the results of particle aging experiments using low particle concentrations in solution. Fig. 2B shows the results of sensing surface aging experiments, where in the starting condition the analogue was hybridized and thereafter a buffer solution (without analogue) was inserted in the measurement chamber.

To be able to distinguish between potential losses of functionality (e.g. denaturing of antibodies or analogues) and potential dissociation of molecules (antibody dissociation from the particles, analogue dissociation from the sensing surface) during the aging experiments, equilibrium-shift experiments were designed, as explained in the body text of the paper.

Figures S5A and B present particle aging experiments performed with different particle concentrations compared to Fig. 2A. The concentrations studied were 2500x dilution to 0.008 mg/mL (Fig. 2A), 150x dilution to 0.07 mg/mL (Fig. S5A), and 12.5x dilution to 0.8 mg/mL (Fig. S5B). The latter two figures show a stable bound fraction as a function of the aging time. This shows that the equilibrium-shift experiments with higher particle concentrations give a lower loss of particle functionality, as neither the direct-assay nor the competition-assay readouts show significant changes as a function of aging time. We attribute the improved stability at high particle concentrations to the occurrence of antibody re-binding. The results suggest that the changes seen at low particle concentrations (2500x dilution, Fig. 2A) are dominantly caused by losses of antibodies and/or losses of blocking molecules from the particles, most likely due to the dissociation of biotin-streptavidin bonds.

Figure S5C shows an equilibrium-shift experiment on the sensing surface, where the solution during the aging experiment contained the analogue molecules with a concentration of 500 pM. The data show that the bound fraction values, with both the direct and competition assay readout, remained constant as a function of the aging time of the surface. This implies that the decrease in bound fraction seen in Fig 2B was mainly due to the dissociation of analogue molecules instead of a loss of functionality of analogue molecules coupled to the sensing surface.

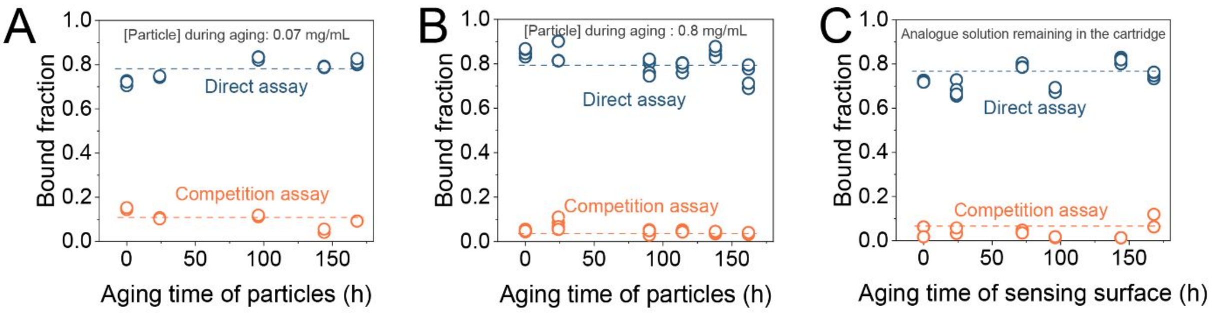

Figure S5. Equilibrium-shift studies of particle and sensing surface aging, with both direct and competition assay f-BPM readouts. Antibody functionalized particles were diluted 150 times to 0.07 mg/mL (A) and 12.5 times to 0.8 mg/mL (B) in 0.5 M NaCl in PBS and incubated during different days at room temperature on a rotatory fin. Particles with different aging times and different dilutions were measured on freshly prepared sensing surfaces, with both direct and competition assay readout. C) Sensing surfaces with cortisol analogue were prepared by adding 500 pM analogue on different days, distributed over a period of 7 days. The analogue solution was not removed, so the flow cells were kept incubating with the analogue solution. On the last day, all the prepared sensing surfaces were washed with 0.5 M NaCl in PBS and then studied with freshly prepared particles, by measuring the bound fraction in a BPM experiment with free particles. Measurements were performed with direct and competition assay readout. Dashed lines are guides to the eye.

SI 6: Number of tethered particles in the BPM sensor over long time spans

Fig. 3A shows a gradual loss of particles in a t-BPM sensor with antibodies on the particles and analogue molecules on the sensing surface. The observed particle loss rate is about 0.39 ± 0.01 % per hour.

Here, we study how the particle loss rate depends on the functionalization of the sensor. Fig. S6A shows results for a completely functionalized sensor, with an average loss rate of about 0.32 ± 0.07 % per hour. Fig. S6B shows results for a sensor with antibodies on the particles and no analogue molecules on the sensing surface, which gives an average particle loss rate of about 1.25 ± 0.30 % per hour. Fig. S6C shows results for a sensor with neither antibodies nor analogue molecules, which gives a particle loss rate of about 0.83 ± 0.15 % per hour.

These experiments show that the particle loss rate depends on the functionalization of the particles and sensing surface, because the loss rate is larger for the control measurements without specific interactions. We attribute this observation to a larger re-tethering probability of particles in case particles have specific antibody-analogue interactions with the sensing surface.

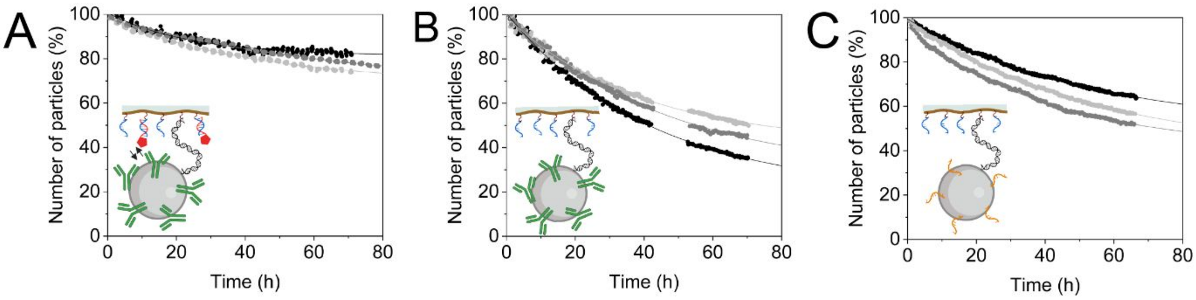

Figure S6. Number of tethered particles in the BPM system over time, with and without specific antibody-analogue interactions. The data points were fitted with an exponential fit, $y = y_0 + a \cdot \exp(-k \cdot t)$ A) t-BPM sensor with antibody on the particles and analogue molecules on the sensing surface. B) t-BPM sensor with antibody on the particles but without analogue molecules on the sensing surface. C) t-BPM sensor with no antibody on the particles nor analogue on the sensing surface. Every experiment was performed with three individual flow cells. The number of particles was normalized to allow a direct comparison of the loss rates of the particle between replicate experiments.

SI 7: Tethered particles before analogue addition

Figures 4A and 4B show distributions of particle motion behavior for a t-BPM sensor with antibodies on the particles and analogue molecules on the sensing surface.

In this Section, we study the distribution characteristics for a t-BPM control experiment, with antibodies on the particles but without analogue molecules on the sensing surface, see Figure S7.

Figure S7A shows that over 80% of the tethered particles are categorized in group 1, which can be attributed to single-tethered particles. The remaining particles are mainly present in group 3, with small motion amplitudes due to multiple tethering or non-specific interactions. Figure S7B shows the particle classification with a color scale based on the number of switching events, as in Figure 4B. The majority of particles shows zero switching events, visualized in a histogram in Figure S7C. Particles with non-zero switching events are attributed to non-specific interactions and signal processing artefacts.

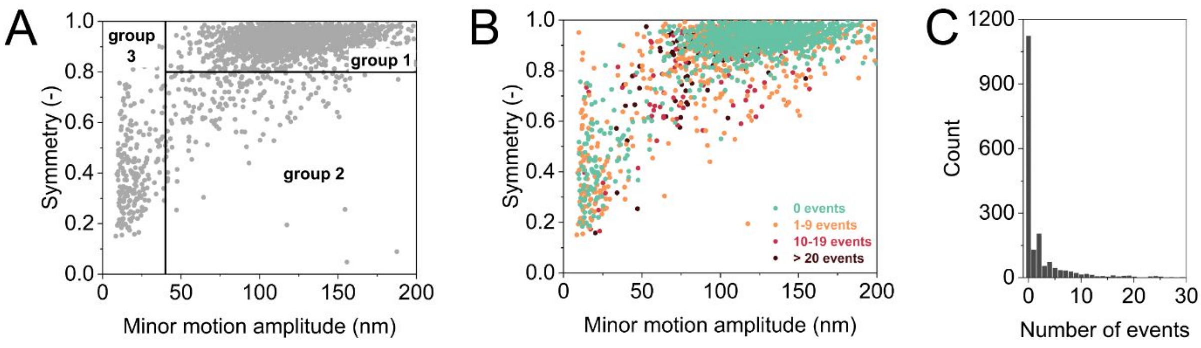

Figure S7. t-BPM experiment before analogue addition. A) Particle motion distribution plot. B) Particle motion distribution plot with colors indicating the number of switching events per particle. C) Histogram of the number of switching events. The average number of switches is 3.4 and the average switching frequency of this sensor is 11.3 mHz.

SI 8: Particle distributions over time for t-BPM negative controls

Figure 4 shows the time dependence of the numbers of particles with different motion patterns (group 1, 2, 3) and with different switching activities, for a t-BPM sensor with antibodies on the particles and analogue molecules on the sensing surface.

In this Section we study the time dependencies in two control experiments: Figure S8A shows data for a t-BPM sensor with antibodies on the particles but without analogue molecules on the sensing surface, and Figure S8B for a t-BPM sensor with neither antibodies on the particles nor analogue molecules on the sensing surface. In both cases, the particles are dominantly in group 1 as shown in Figures S8A(I) and S8B(I), and most particles show zero switching events, see Figures S8A(II) and S8B(II).

Figures S8A(III) and B(III) display the number of non-switching particles across the three groups. The number of particles with zero switching events decreases over time. This is attributed to the loss of particles, as discussed in Figure S6.

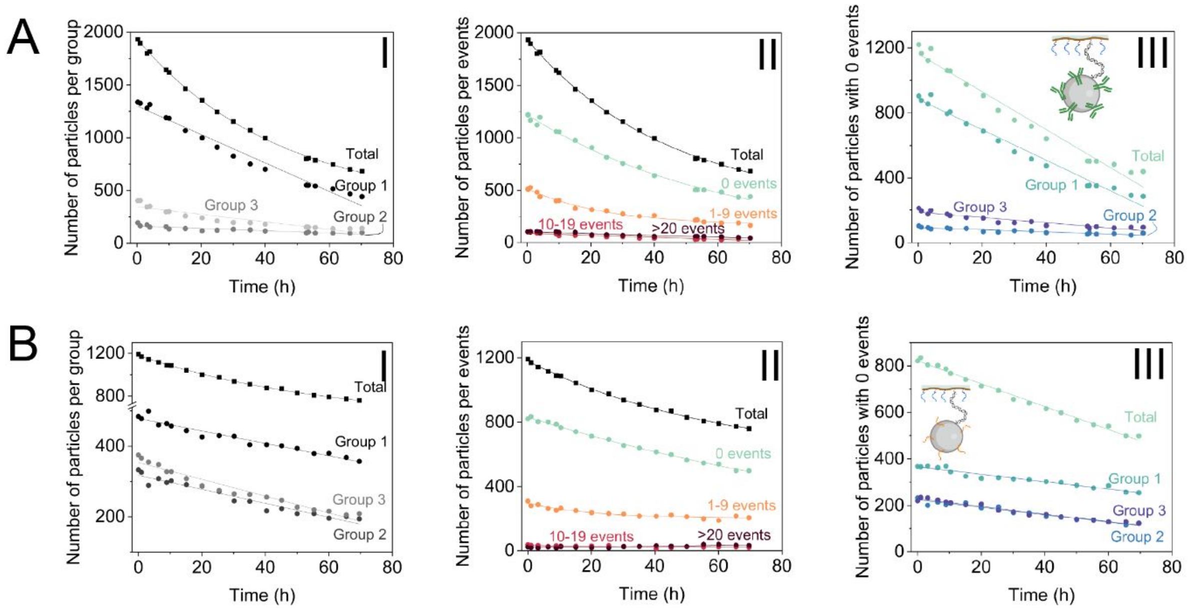

Figure S8. Number of particles tracked over time for t-BPM sensors without specific antibody-analogue interactions. A) t-BPM sensor with antibodies on the particles and without analogue molecules on the sensing surface. B) t-BPM sensor without antibodies on the particles and without analogue molecules on the sensing surface. I) Number of particles classified into the three different groups based on their motion patterns, plotted as a function of time. II) Number of particles classified in terms of the number of switching events per particle (0, 1-9, 10-19, or more than 20 events), plotted as a function of time. III) Number of particles showing zero switching events, classified in terms of their motion patterns (group 1, group 2, group 3), plotted as a function of time. Lines are guides to the eye.

References

(1) Bergkamp, M. H.; Cajigas, S.; van IJzendoorn, L. J.; Prins, M. W. J. High-Throughput Single-Molecule Sensors: How Can the Signals Be Analyzed in Real Time for Achieving Real-Time Continuous Biosensing? ACS Sens 2023, 8 (6), 2271–2281. https://doi.org/10.1021/acssensors.3c00245.

(2) Lin, Y.; Vermaas, R.; Yan, J.; Jong, A. M. De; Prins, M. W. J. Click-Coupling to Electrostatically Grafted Polymers Greatly Improves the Stability of a Continuous Monitoring Sensor with Single-Molecule Resolution. ACS Sens 2021, 6 (5), 1980–1986. https://doi.org/https://doi.org/10.1021/acssensors.1c00564.

(3) Yan, J.; Van Smeden, L.; Merkx, M.; Zijlstra, P.; Prins, M. W. J. Continuous Small-Molecule Monitoring with a Digital Single-Particle Switch. ACS Sens 2020, 5 (4), 1168–1176. https://doi.org/https://dx.doi.org/10.1021/acssensors.0c00220.

(4) Visser, E. W. A.; Yan, J.; Van IJzendoorn, L. J.; Prins, M. W. J. Continuous Biomarker Monitoring by Particle Mobility Sensing with Single Molecule Resolution. Nat Commun 2018, 9 (1), 1–10. https://doi.org/10.1038/s41467-018-04802-8.

(5) Vu, C.; Lin, Y.; Haenen, S. R. R.; Marschall, J.; Hummel, A.; Wouters, S. F. A.; Raats, J. M. H.; Jong, A. M. De; Yan, J.; Prins, M. W. J. Real-Time Immunosensor for Small-Molecule Monitoring in Industrial Food Processes. Anal Chem 2023, 95, 7950–7959. https://doi.org/10.1021/acs.analchem.3c00628.

(6) Bergkamp, M. H.; Cajigas, S.; van IJzendoorn, L. J.; Prins, M. W. J. Real-Time Continuous Monitoring of Dynamic Concentration Profiles Studied with Biosensing by Particle Motion. Lab Chip 2023, 23 (20), 4600–4609. https://doi.org/10.1039/d3lc00410d.

(7) Visser, E. W. A.; Van IJzendoorn, L. J.; Prins, M. W. J. Particle Motion Analysis Reveals Nanoscale Bond Characteristics and Enhances Dynamic Range for Biosensing. ACS Nano 2016, 10 (3), 3093–3101. https://doi.org/10.1021/acsnano.5b07021.

(8) Bergkamp, M. H.; Van IJzendoorn, L. J.; Prins, M. W. J. Real-Time Detection of State Transitions in Stochastic Signals from Biological Systems. ACS Omega 2021, 6 (27), 17726–17733. https://doi.org/https://doi.org/10.1021/acsomega.1c02498.

(9) Buskermolen, A. D.; Lin, Y. T.; van Smeden, L.; van Haaften, R. B.; Yan, J.; Sergelen, K.; de Jong, A. M.; Prins, M. W. J. Continuous Biomarker Monitoring with Single Molecule Resolution by Measuring Free Particle Motion. Nat Commun 2022, 13 (1), 6052. https://doi.org/10.1038/s41467-022-33487-3.

(10) Buskermolen, A. D.; Michielsen, C. M. S.; de Jong, A. M.; Prins, M. W. J. Towards Continuous Monitoring of TNF-α at Picomolar Concentrations Using Biosensing by Particle Motion. Biosens Bioelectron 2024, 249, 115934. https://doi.org/10.1016/J.BIOS.2023.115934.

(11) Van Smeden, L.; Saris, A.; Sergelen, K.; De Jong, A. M.; Yan, J.; Prins, M. W. J. Reversible Immunosensor for the Continuous Monitoring of Cortisol in Blood Plasma Sampled with Microdialysis. ACS Sens 2022, 7 (10), 3041–3048. https://doi.org/10.1021/acssensors.2c01358.
